# Structural Changes and Proton Transfer in Cytochrome *c* Oxidase

**DOI:** 10.1038/srep12047

**Published:** 2015-08-27

**Authors:** Jóhanna Vilhjálmsdóttir, Ann-Louise Johansson, Peter Brzezinski

**Affiliations:** 1 Department of Biochemistry and Biophysics, The Arrhenius Laboratories for Natural Sciences, Stockholm University, SE-106 91 Stockholm, Sweden

## Abstract

In cytochrome *c* oxidase electron transfer from cytochrome *c* to O_2_ is linked to transmembrane proton pumping, which contributes to maintaining a proton electrochemical gradient across the membrane. The mechanism by which cytochrome *c* oxidase couples the exergonic electron transfer to the endergonic proton translocation is not known, but it presumably involves local structural changes that control the alternating proton access to the two sides of the membrane. Such redox-induced structural changes have been observed in X-ray crystallographic studies at residues 423–425 (in the *R. sphaeroides* oxidase), located near heme *a*. The aim of the present study is to investigate the functional effects of these structural changes on reaction steps associated with proton pumping. Residue Ser425 was modified using site-directed mutagenesis and time-resolved spectroscopy was used to investigate coupled electron-proton transfer upon reaction of the oxidase with O_2_. The data indicate that the structural change at position 425 propagates to the D proton pathway, which suggests a link between redox changes at heme *a* and modulation of intramolecular proton-transfer rates.

Cytochrome *c* oxidase (Cyt*c*O) is a membrane-bound protein complex, which utilizes the free energy released during electron transfer from cytochrome *c* to O_2_, to proton pumping across the membrane. The initial electron acceptor in Cyt*c*O is Cu_A_, which is composed of two copper ions. In the *aa*_3_-type Cyt*c*O from *Rhodobacter* (*R*.) *sphaeroides* electrons are transferred from Cu_A_ consecutively to heme *a* and then to the catalytic site, which is composed of heme *a*_3_ and Cu_B_. In this Cyt*c*O the proton-pumping stoichiometry is on average about one proton pumped, from the negative (*n*) side to the positive (*p*) side of the membrane, per electron transferred to O_2_. The catalytic site is buried within the membrane-spanning part of the protein, at a distance of ~2/3 of the membrane thickness from the *n*-side of the membrane. Protons that are involved in reduction of O_2_ to H_2_O (O_2_ + 4H^+^ + 4e-→2H_2_O) are transferred through two proton pathways linking the *n*-side surface with the catalytic site. One of these pathways, denoted by the letter D, is also used for transfer of all protons that are pumped across the membrane. The branching point where protons are directed from the D pathway either to the catalytic site or an acceptor site for the pumped protons (see below) is a highly conserved Glu residue (Glu286) (Fig. 1a), located at a distance of ~25 Å from the *n*-side surface (for recent publications on the structure and function of the Cyt*c*Os, see e.g.^1^,^2^,^3^,^4^,^5^,^6^,^7^,^8^,^9^,^10^,^11^,^12^,^13^).

A redox-driven proton pump must fulfill a number of functional requirements, which are reflected in the structural design of the protein. First, proton pumping across the membrane requires specific proton-conduction pathways spanning the entire distance from the *n* to the *p* side surface. However, the proton pathway(s) can not be open to both sides of the membrane simultaneously over time scales of the proton transfer because this arrangement would result in a proton short circuit across the membrane. Consequently, proton transfer must be controlled by the protein. The control element could, for example, be composed of a protonatable residue or a cluster of residues that can rapidly exchange protons with either the *n* or *p* side of the membrane. This protonatable site is often referred to as the “proton-loading site”, PLS^14^,^15^,^16^. One possible general design of the proton pump would be to link the alternating proton access to and from the PLS, to intramolecular electron transfer such that the free energy from the electron transfer would be conserved by changing the p*K*_a_ of the PLS. The p*K*_a_ of the PLS would be high (relative to the solution pH) when in contact with the proton-input side and low when in contact with the proton-output side.

On the basis of the general considerations outlined above, it is likely that changes in the redox state of the metal cofactors result in local structural changes that control the proton access to the two sides of the membrane. Because the proton-tunneling distance is on the order of ~1 Å, the rate of proton transfer can easily be modulated by minor structural changes^17^. When considering the *aa*_3_ oxidases, early structures of the oxidized and reduced forms of the *Paracoccus denitrificans* Cyt*c*O revealed essentially no structural changes^18^. Results from more recent amide hydrogen-deuterium exchange Mass Spectrometric studies^19^,^20^ and from X-ray crystallographic studies of Cyt*c*O from *R. sphaeroides* and bovine heart indicate that structural changes upon reduction of the oxidized Cyt*c*O do occur and that these changes are functionally relevant^8^,^21^,^22^,^23^,^24^,^25^,^26^ (possible reasons from differences between results from the early and more recent studies are discussed in^8^).

A segment of the protein that has attracted particular interest is that at helix X, located between the hemes. Here, a significant change in side chain configuration was observed for residue Ser425 (Ser382 in the bovine heart mitochondrial Cyt*c*O), ~7 Å from the heme *a* iron (see Fig. 1a, the change in position is shown in^8^,^22^). Similar changes in structure were observed with both the *R. sphaeroides*^8^,^21^,^22^ and mitochondrial Cyt*c*Os^23^,^24^. In the mitochondrial oxidase these changes were suggested to be part of the proton pumping mechanism by modulation of proton transfer through the H pathway^27^. In the *R. sphaeroides* Cyt*c*O the redox-induced changes were suggested to modulate the proton access via the K and D proton pathways, respectively^8^. Interestingly, the redox induced changes were also linked to changes in water structure, presumably modulating the proton access from Glu286 to the catalytic site. In other words, these results suggest a link between re-allocation of the Ser425 side chain and proton transfer from the D pathway^8^. These redox-induced structural changes should also modulate proton transfer to the PLS because, as outlined above, an alternating proton access to the PLS must be part of the proton-pumping mechanism.

The distance between Ser425 and Glu286 is ~15 Å. The location of the PLS is not known, but experimental and theoretical data suggest that it is found in a protein segment around one of the His ligands of Cu_B_ or the propionic acids of heme *a*_3_^28^,^29^,^30^,^31^,^32^, most likely including a cluster of residues in this region^14^, all found at a distance of 13–20 Å from Ser425.

On the basis of the discussion above, we reasoned that if the observed structural changes around Ser425 are mechanistically relevant, then a structural modification in the general vicinity of Ser425 should affect proton transfer through the D pathway. Consequently, we used site-directed mutagenesis to replace Ser425 by e.g. Ala, and then investigated the kinetics of electron and proton transfer reactions, associated with O_2_ reduction, in parts of the reaction cycle that are linked to proton pumping via the D pathway. To study these reactions, we measured absorbance changes of the Cyt*c*O cofactors as well as that of a pH-sensitive dye in solution after flash photolysis of the reduced Cyt*c*O-CO complex in the presence of O_2_. After dissociation of the CO ligand, O_2_ binds to heme *a*_3_, which is followed in time by step-wise electron transfer from all four redox sites. This approach allows observation of the formation and decay of intermediate states that are populated during reduction of O_2_ to H_2_O, catalyzed by Cyt*c*O (see Fig. 1b and e.g.^33^,^34^,^35^ for a more detailed description of the technique). The data show that at neutral pH the Ser425Ala structural variant displayed the same electron and proton-transfer rates as the wild-type Cyt*c*O. However, at low proton concentrations (pH>9), rapid proton uptake to Glu286 was maintained at high pH, indicating that the structural modification at position 425 resulted in changes of a reaction step that is rate limiting for proton transfer through the D proton pathway.

## Results

To investigate the function of the Ser425Ala variant, we first studied internal electron transfer between the hemes in the absence of oxygen^36^. A state of the Cyt*c*O was prepared in which heme *a*_3_ and Cu_B_ are reduced while heme *a* and Cu_A_ are oxidized, and carbon monoxide is bound to heme *a*_3_. The CO ligand stabilizes the reduced state of heme *a*_3_. Consequently, after light-induced dissociation of CO (Fig. 2, rapid absorbance increase at *t* = 0) the electron at heme *a*_3_ equilibrates with heme *a*, which is seen as a decrease in absorbance with a time constant of 2.2 ± 0.2 μs (SD of 10 traces) with the wild-type Cyt*c*O. As seen in the Figure, this absorbance decrease was faster with the Ser425Ala variant Cyt*c*O (τ = 1.0 ± 0.2 μs, SD of 10 traces).

**Figure 1:**
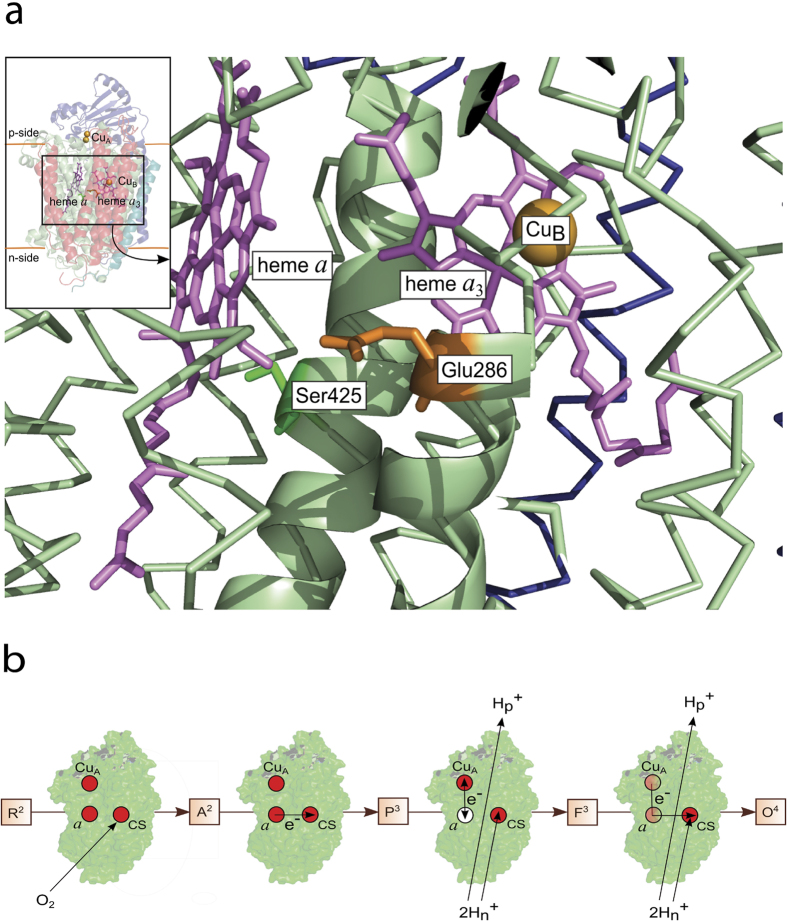
Structure and function of Cyt*c*O. (**a**) The inset shows the overall structure (PDB entry 1M56) of Cyt*c*O from *R. sphaeroides*, the redox-active cofactors involved in electron transfer and the approximate position of the membrane. The main figure shows the relative positions of Ser425, Glu286, hemes *a* and *a*_3_ as well as Cu_B_. The figure was prepared using PyMOL^73^. (**b**) The reactions studied in this work (see text for detailed description, the superscripts in the square boxes refer to the number of electrons at the catalytic site).

Next, we studied the reaction of the four-electron reduced Cyt*c*O with O_2_. The Cyt*c*O was reduced and incubated under CO atmosphere to form the Cyt*c*O-CO complex. The sample was then rapidly mixed with an O_2_-saturated solution after which the CO ligand was dissociated with a laser flash. The mixing allowed O_2_ to bind to heme *a*_3_ thereby initiating the oxidation of the Cyt*c*O. As seen previously (e.g.^37^), with the wild-type Cyt*c*O, at 445 nm (Fig. 3a), the increase in absorbance at *t* = 0 is associated with dissociation of the CO ligand yielding the reduced Cyt*c*O (state **R**^2^ in Fig. 1b, the superscript refers to the number of electrons at the catalytic site). It is followed in time by an absorbance decrease with a time constant of ~10 μs associated with binding of O_2_ (at 1 mM O_2_) forming state **A**^**2**^. In the next step the electron at heme *a* is transferred to the catalytic site with a time constant of ~40 μs forming the peroxy state called **P**^**3**^, which is associated with a small decrease in absorbance at 445 nm. This electron transfer is also seen as a decrease in absorbance at 580 nm (Fig. 3b).

The electron transfer from heme *a* to the catalytic site triggers proton uptake from solution (Fig. 4) to the catalytic site, which at neutral pH displays a time constant of ~100 μs at pH 7.4. The proton uptake yields the ferryl state, **F**, at the catalytic site, which is seen as an increase in absorbance at 580 nm (Fig. 3b). The proton uptake associated with the **P3** → **F**^**3**^ reaction is also linked to fractional electron transfer from Cu_A_ to heme *a*, seen as an increase in absorbance at 830 nm with a time constant of ~100 μs (Fig. 3c, oxidation of Cu_A_). In the final step of the reaction, the electron in the Cu_A_—heme *a* equilibrium is transferred to the catalytic site forming the oxidized Cyt*c*O (state **O**^**4**^) with a time constant of ~1 ms at neutral pH. This reaction is seen as a decrease in absorbance at 445 nm and at 580 nm (Fig. 3a,b) and an increase in absorbance at 830 nm (Fig. 3c, oxidation of Cu_A_). A comparison of the traces recorded at neutral pH (Fig. 3) shows only minor differences between the wild-type and Ser425Ala variant Cyt*c*Os, except that the **P**^**3**^ **→** **F**^**3**^ rate was slightly increased from ~1.1·10^4^s^−1^ to ~1.5·10^4^s^−1^ (Fig. 5a) and the extent of electron transfer from Cu_A_ to heme *a* (Fig. 3c) was slightly larger with the Ser425Ala Cyt*c*O than with the wild-type Cyt*c*O, which indicates that at pH ~7 the midpoint potential of heme *a* was slightly higher with the Ser425Ala than with the wild-type Cyt*c*O.

Results from earlier studies have shown that functional differences caused by minor changes in structure may be revealed at low proton concentrations (e.g.^38^). Consequently, we investigated the reaction steps that are linked to proton uptake (and pumping) during reaction of the reduced Cyt*c*O with O_2_ also at high pH (Figs 3 and 5). Earlier studies with the wild-type Cyt*c*O have shown that the **P3** → **F**^**3**^ reaction rate is approximately pH independent up to pH ~9 and then it decreases with increasing pH at higher pH (Fig. 5a). In contrast, with the Ser425Ala variant, the **P3** → **F**^**3**^ rate displayed a much weaker pH dependence, the rate constant dropped by less than a factor of ~2 from ~1.3·10^4^ s^−1^ at pH 6 to ~7.8·10^3^ s^−1^ at pH 10.5 (Fig. 5a, the rate shown in this figure was obtained by fitting data at both 580 nm (**P3** → **F**^**3**^) and at 830 nm (Cu_A_ oxidation)). The extent of electron transfer from Cu_A_ to heme *a* was significantly larger with the Ser425Ala than with the wild-type Cyt*c*O (Fig. 5c). We also measured the **P3** → **F**^**3**^ rate with two other structural variants at the Ser425 position, Ser425Thr and Ser425Val, but only at 3 pH values, *i.e.* 6, 8 and 10. Qualitatively, the same behavior was observed as with the Ser425Ala variant (see Fig. 5a,b). The **F** ^**3**^→ **O**^**4**^ rate constant displayed similar pH dependence with the Ser425Ala as with the wild-type Cyt*c*O.

To investigate whether or not the Ser425Ala variant pumps protons, the Cyt*c*O was reconstituted in lipid vesicles with the pH dye phenol red added to the outside of the vesicles (see “Materials and Methods”) and the dye absorbance changes were measured as a function of time during Cyt*c*O turnover. The data showed that the Ser425Ala variant pumps protons with about the same stoichiometry as the wild-type Cyt*c*O (Fig. 6).

## Discussion

We have investigated the effect of a structural modification in a segment of Cyt*c*O recently shown to be involved in redox-induced structural changes^8^,^21^,^22^,^23^,^24^. The activity of the Ser425Ala variant was about the same as that of the wild-type Cyt*c*O. Furthermore, at neutral pH internal electron and proton-transfer as well as proton uptake from solution displayed the same rates as with the wild-type Cyt*c*O. However, significant differences were observed between the Ser425Ala variant and the wild-type Cyt*c*O when the proton supply was limited (i.e. at high pH).

**Figure 2:**
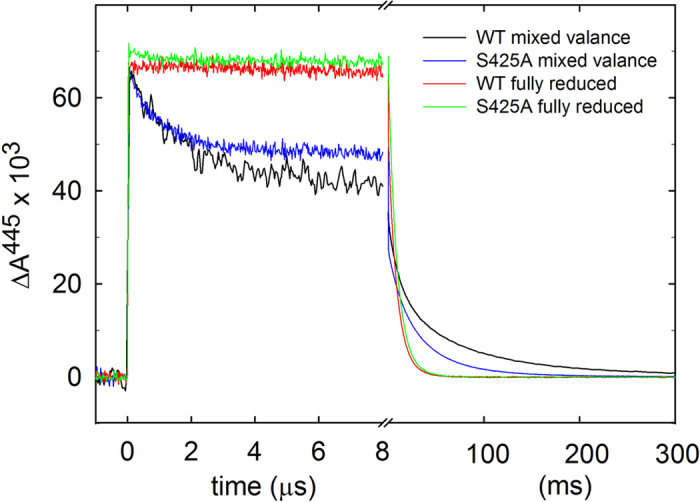
Absorbance changes associated with internal electron transfer in the absence of O_2_. A sample of Cyt*c*O with heme *a*/Cu_A_ oxidized, heme *a*_3_/Cu_B_ reduced and CO bound to heme *a*_3_ was prepared. The Cyt*c*O was illuminated with a short laser flash, which resulted in dissociation of the CO ligand, seen as an increase in absorbance at *t* = 0. The rapid decrease in absorbance is associated with electron transfer from heme *a*_3_ to heme *a* (τ ≅ 2.2 ± 0.2 μs and 1.0 ± 0.2 μs with the wild-type and Ser425Ala mutant Cyt*c*Os, respectively). The absorbance then decreased slowly to reach the original level at *t* < 0 with time constants of ~65 ms and ~50 ms for the wild-type Ser425Ala Cyt*c*Os, respectively. Experimental conditions: the Cyt*c*O concentration was ~0.7 μM, 100 mM BIS-TRIS propane, pH 8.5, 0.05% DDM, 0.1 mM EDTA at ~22 °C and ~1.4 mM CO (at 140 kPa). In cases where heme *a* became fractionally reduced, the enzyme solution was titrated with anaerobic ferricyanide until heme *a* became re-oxidized as determined from the optical absorption spectra. Ascorbate (2 mM) and the redox mediator hexamine-ruthenium chloride (1 μM) were added to fully reduce the Cyt*c*O. The traces have been scaled to 1 μM reacting Cyt*c*O.

**Figure 3:**
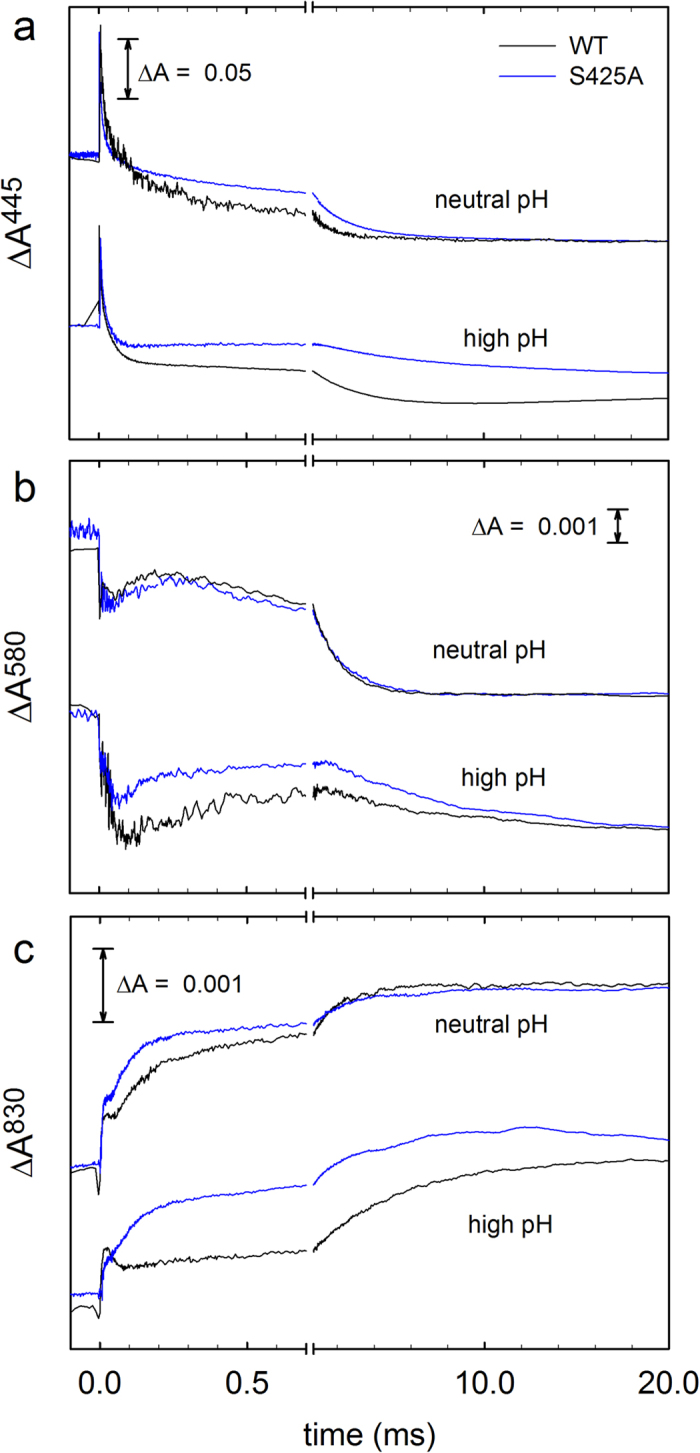
Absorbance changes associated with reaction of the reduced Cyt*c*O with O_2_. At all wavelengths the initial absorbance change at *t* = 0 is associated with dissociation of the CO ligand. (**a**) At 445 nm the main contribution is from the heme groups. After CO dissociation, the absorbance decreases as a result of binding of O_2_ (τ ≅ 10 μs), followed by formation of **P**^**3**^ (τ ≅ 40 μs) and oxidation of the Cyt*c*O, i.e. formation of state **O**^**4**^ (τ ≅ 1.0 ms and τ ≅ 1.5 ms for the wild-type and Ser425Ala Cyt*c*Os, respectively, at neutral pH). Neutral and high pH are pH 7.0 (WT)/6.5 (S425A) and pH 10 (WT)/10.5 (S425A), respectively. (**b**) At 580 nm the absorbance decrease in the time range 5 μs–100 μs is associated with formation of the **P**^**3**^ state. The increase in absorbance at neutral pH in the range 100–300 μs is associated with the **P**^**3**^ **→** **F**^**3**^ reaction with time constants of ~90 μs and ~60 μs for the wild-type and Ser425Ala Cyt*c*Os, respectively. The final absorbance decrease is associated with the **F**^**3**^ **→** **O**^**4**^ reaction. Neutral and high pH are pH 7.4 (WT)/6.5 (S425A) and pH 10 (WT)/10.5 (S425A), respectively. Wild-type data are from^43^. (**c**) At 830 nm the main contribution to the absorbance changes is from Cu_A_ where an increase in absorbance is associated with oxidation of this redox center. Neutral and high pH are pH 7.4 (WT)/6.5 (S425A) and pH 10.4 (WT)/10.5 (S425A), respectively. Experimental conditions after mixing: Cyt*c*O concentration 0.3–1.3 μM, 0.05% DDM, 100 μM EDTA at pH 7.5, 1 mM O_2_ at ~22 °C. The mixing ratio was 1:5 with oxygen-saturated buffer solutions (100 mM): MES at 6.5; HEPES at pH 7, 7.4 and 7.5; CAPS, pH 10, 10.4 and10.5. The traces have been scaled to 1 μM reacting enzyme.

As seen in Fig. 2, after CO dissociation from the mixed-valence Cyt*c*O (i.e. Cyt*c*O in which heme *a*_3_ and Cu_B_ are reduced while heme *a* and Cu_A_ are oxidized) a rapid decrease in absorbance at 445 nm was observed. With the *R. sphaeroides* Cyt*c*O, this relatively large absorbance change is associated with electron transfer from heme *a*_3_ to heme *a*^36^,^39^, which was faster with the Ser425Ala Cyt*c*O (τ ≅ 1.0 μs) than with the wild-type Cyt*c*O (τ ≅ 2.2 μs). More recent data suggest that this electron transfer is controlled by a structural relaxation linked to CO dissociation from Cu_B_^40^, which transiently binds the CO after its dissociation from heme *a*_3_. Thus, the data indicate that a structural change around Ser425 modulates the Cu_B_ environment. This observation is consistent with the observed changes around Glu286 as a result of the Ser425Ala replacement (see discussion below) because one of the Cu_B_ ligands, His284, is separated by only a single residue from Glu286 and a link between changes at Glu286 and at the catalytic site have been observed in the past^41^.

The data in Fig. 5c show that during the **P**^**3**^ **→** **F**^**3**^ reaction a significantly larger fraction of heme *a* becomes reduced with the Ser425Ala (70%) than with the wild-type Cyt*c*O (~55%, see also^37^). This electron is transferred from Cu_A_ and the fraction electron transfer reflects the Cu_A_—heme *a* equilibrium constant, i.e. the difference in midpoint potentials between the two sites. Assuming that the effect of the amino-acid replacement is on heme *a* and not Cu_A_ (because of the much shorter distance from Ser425 to heme *a* than to Cu_A_), the difference observed in the current study is consistent with an increase in the heme *a* midpoint potential of ~20 mV.

**Figure 4:**
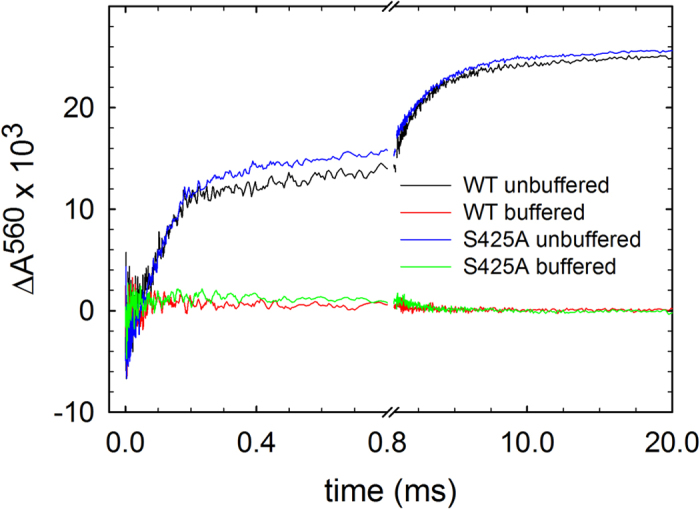
Absorbance changes of the dye phenol red associated with proton uptake during O_2_ reduction. The same reaction as in Fig. 3 was studied, but at 560 nm where the main contribution is from the dye. The signal shown is a difference between that obtained without buffer and that obtained with buffer. Experimental conditions after mixing: ~0.6 μM reacting enzyme in 100 mM KCl, pH 7.8, 0.05% DDM, 40 μM phenol red, 100 μM EDTA, 1 mM O_2_ at ~22 °C. The measurements were then repeated in the presence of 0.1 M HEPES (without KCl) and these absorbance changes were subtracted from those obtained with the un-buffered solution yielding absorbance changes that were only associated with proton uptake. The shown traces are average of 15 experiments.

During reaction of the reduced Cyt*c*O with O_2_, the first step that is associated with proton uptake from the *n* side (and proton release to the *p* side of the membrane) is the **P**^**3**^ **→** **F**^**3**^ reaction^42^ (τ ≅ 100 μs, see Fig. 4). Because an electron is transferred to the catalytic site during the preceding step, **A**^**2**^ **→** **P**^**3**^ (τ ≅ 40 μs), the **P**^**3**^ **→** **F**^**3**^ reaction involves only proton transfer, i.e. there is no internal electron transfer over the time scale of this reaction. As discussed in more detail below, the observed p*K*_a_ in the pH dependence of the **P**^**3**^ **→** **F**^**3**^ rate presumably reflects that of Glu286, which is a component of the proton-transfer pathway. Consequently, changes in this p*K*_a_ value as a result of the Ser425Ala structural modification presumably reflect effects on the Glu286 p*K*_a_.

According to a model that we have developed previously to interpret kinetic data from measurements of the **P**^**3**^ **→** **F**^**3**^ reaction^38^,^43^, proton transfer through the D pathway involves a rapid (<100 μs) equilibrium of Glu286 with the *n*-side solution and the rate-limiting step is proton transfer from Glu286 to the catalytic site. Thus, according to this model the pH dependence of the **P**^**3**^ **→** **F**^**3**^ rate (*k*_PF_) reflects the p*K*_a_ of Glu286 (p*K*_E286_) as outlined in Eq. 1 below:


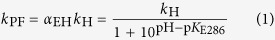


where α_EH_ is the fraction protonated Glu286 and *k*_H_ is the proton-transfer rate from Glu286 to the catalytic site. With the wild-type *R. sphaeroides* Cyt*c*O the value of p*K*_E286_ was found to be 9.4^43^,^44^. In the following sections we argue that the p*K*_a_ of Glu286 in the Ser425Ala variant is higher than that in the wild-type Cyt*c*O.

As already indicated above, the Glu286 residue is located at the end of the D pathway from where protons are transferred either to the catalytic site or toward the PLS. The proton flux to the two sites is presumably modulated by the Glu286 side chain conformation^10^. Furthermore, the Glu286 connectivity to the catalytic site could also be regulated by redox-induced reallocation of water molecules between the two sites^21^. The model in Eq. 1 is a simplistic description of the problem because in the **P**^**3**^ **→** **F**^**3**^ reaction two protons are transferred simultaneously via Glu286, one to the PLS and one to the catalytic site. The mechanism by which the Glu286 controls the proton flux to the two acceptors is not understood^45^,^46^, but on the basis of results from earlier studies^8^,^10^,^47^,^48^,^49^,^50^,^51^,^52^,^53^ we speculate on the involvement of Glu286. It is likely that during each of the four reactions steps that are linked to proton pumping (such as e.g. the two reaction steps, **P**^**3**^ **→** **F**^**3**^ and **F**^**3**^ **→** **O**^**4**^, studied here) the Glu side chain would switch from its relaxed configuration, where it is in contact with the *n*-side of the membrane, to a transient conformation from where it could transfer a proton to the PLS or the catalytic site. According to a previously presented model, the p*K*_a_s are different in these two configurations^44^,^51^,^54^,^55^. The changes in configuration and p*K*_a_ would be repeated four times during each reaction cycle, i.e. for each transition that is linked to proton pumping. According to the model^44^,^51^,^54^,^55^ the regulatory function of Glu286 was included by assuming two conformations of the Glu286 side chain, in rapid equilibrium (≪100 μs), with two different p*K*_a_ values:


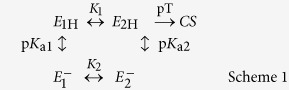


where *E*_1_ and *E*_2_ are the two conformations of Glu286 (*E*_x_^−^ and *E*_xH_, deprotonated and protonated states, respectively) and p*K*_a1_ and p*K*_a2_ are the p*K*_a_ values of Glu286 in these two states. *K*_1_ and *K*_2_ are equilibrium constants and pT indicates proton transfer from state *E*_2H_ to the catalytic site (*CS*). The apparent (observed) p*K*_a_ value of Glu286, p*K*_app_, (defined as the one half of the maximum [E_2H_] value) is:


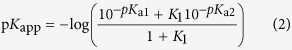


Here, in the E_1_ configuration the side chain would be in contact with the D pathway, i.e. the *n-*side of the membrane. In E_2_ the side chain would be in contact with the catalytic site and presumably also with the PLS although the latter is not explicitly included in the model in Scheme 1 (discussed in more detail below). According to this model the observed p*K*_a_ value in measurements of e.g. the **P**^**3**^ **→** **F**^**3**^ kinetics also reports the equilibrium constant between the two Glu286 side chain configurations^44^,^51^,^55^. It should be noted that the model outlined in Scheme 1 qualitatively describes the scenario in each of the four reaction steps that are linked to proton pumping (four consecutive electron transfers from cytochrome *c* to O_2_ occur for each turnover). However, the actual values of the parameters may vary slightly between the four steps, e.g. due to differences in the overall charge distribution within the protein and the presence of an electrochemical potential across the membrane.

Results from earlier functional studies of a large number of structural variants of *aa*_3_ Cyt*c*Os showed that a number of specific single or double amino-acid residue replacements within the entire span of the D pathway result in Cyt*c*O that is active (i.e. reduces O_2_ to H_2_O), but in which proton pumping is completely blocked^38^,^55^,^56^,^57^,^58^,^59^,^60^,^61^. Detailed kinetic studies of specific electron- and proton-transfer reactions in these Cyt*c*O variants revealed an altered pH dependence of the **P**^**3**^ **→** **F**^**3**^ reaction, interpreted in terms of an altered apparent p*K*_a_ of Glu286^44^,^51^. We suggested that the molecular explanation for this p*K*_app_ shift was a change in the equilibrium constant between the two Glu286 side-chain conformations (see Scheme 1)^44^,^51^. We note that a change in the *K*_1_ and *K*_2_ values in Scheme 1. a change in equilibrium between the two states) would result in a change in p*K*_app_ even if p*K*_a1_ and p*K*_a2_ were unaltered. Furthermore, a structural alteration that changes the equilibrium constant between the two Glu286 positions would most likely also alter the relative rates of reactions that follow in time after the E_1_ to E_2_ switch. When this switch to the E_2_ configuration has occurred there are three competing reactions: (*i*) proton transfer to the catalytic site, (*ii*) proton transfer to the PLS and (*iii*) the structural relaxation from state E_2_ back to E_1_. As discussed earlier^51^,^54^, proton pumping requires that either the proton to PLS is transferred first or, if proton transfer to the catalytic site occurs first, the free energy of this reaction is conserved in a local structural change that drives a consecutive protonation of the PLS. The model outlined in Scheme 1 is a minimum to describe the pH dependence of e.g. the **P**^**3**^ **→** **F**^**3**^ reaction and, as already mentioned above, it does not explicitly include proton transfer to the PLS because this proton transfer is not studied in the experiments that the model is based on. Nevertheless, protonation of the PLS requires that the relative rates of the three reactions (*i*)—(*iii*) above fulfill the requirement that PLS becomes protonated before the structural relaxation takes place. Proton pumping can tolerate a change in all three rates constants provided that their relative values are such that PLS receives a proton. If this requirement is not fulfilled, which is suggested for the uncoupled mutants discussed above, PLS does not receive a proton.

In the context of the above discussion we also note that the change in observed Glu286 p*K*_a_ and absence of proton pumping would both be result of structural changes around the Glu286 site. However, these two effects must not necessarily be interdependent as the p*K*_a_ reflects the equilibrium constant between conformations 1 and 2 in Scheme 1, while the proton-pumping efficiency would be determined by the relative rates of events (*i*)-(*iii*). Several other models explaining the lack of proton pumping in e.g. the Asn139Asp variant Cyt*c*O have been presented (see e.g.^51^). Here, we focus on the model outlined in Scheme 1 above.

**Figure 5:**
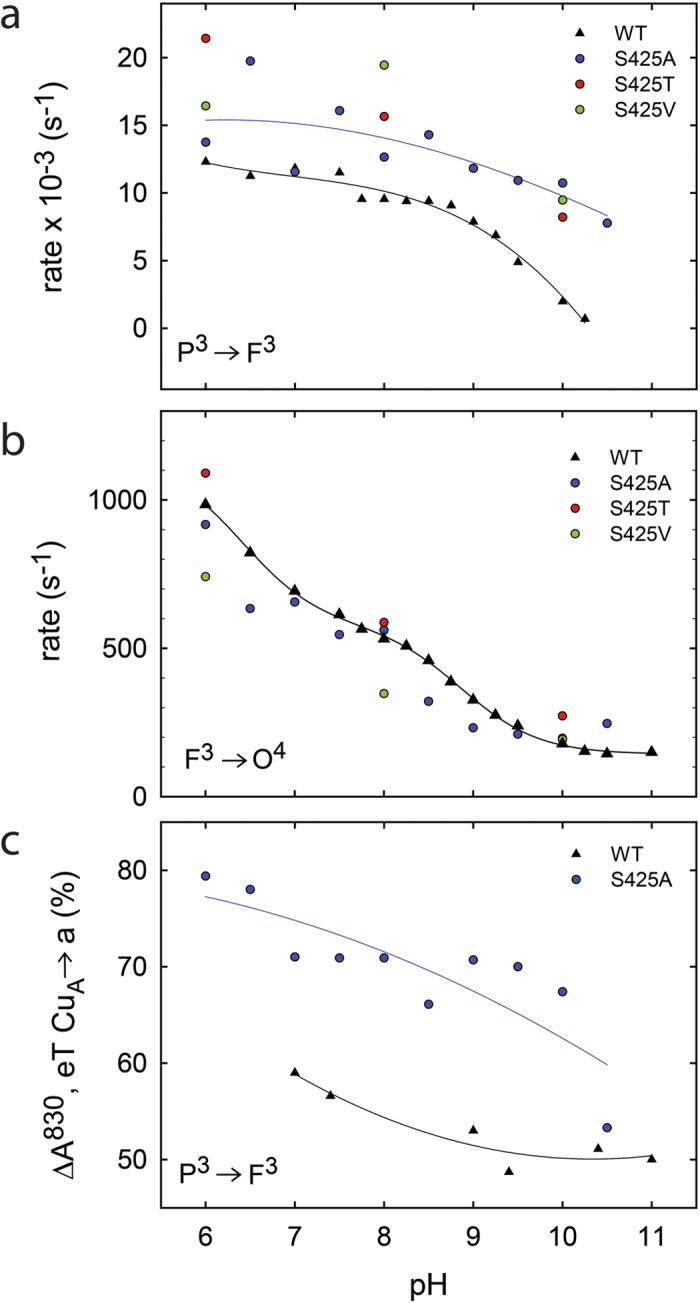
pH dependence of reaction steps associated with proton uptake. (**a**) The **P**^**3**^ **→** **F**^**3**^ reaction, where the rate constants are derived from absorbance changes at 580 nm and at 830 nm. (**b**) The **F**^**3**^ **→** **O**^**4**^ reaction, where the rate constants are derived from absorbance changes at 445 nm, 580 nm and 830 nm. (**c**) Amplitude of the 830-nm absorbance change (electron transfer Cu_A_ —> heme *a*) in percent of the total change at this wavelength (wild-type data are from^3^,^58^).

**Figure 6:**
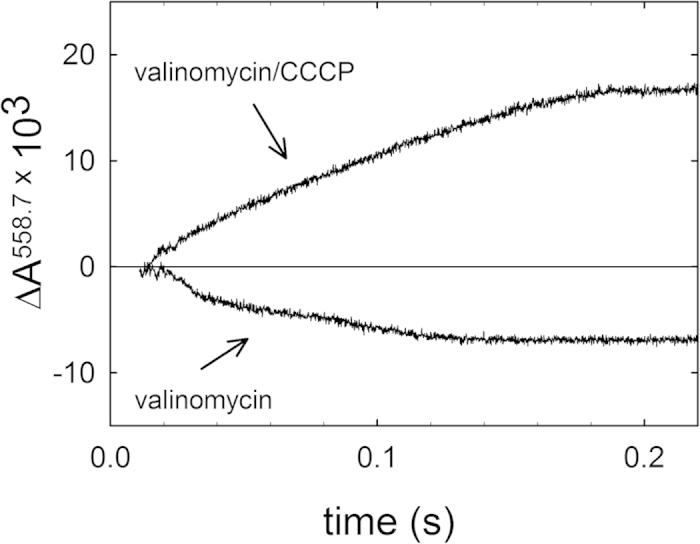
Absorbance changes of the dye phenol red at 558.7 nm associated with proton pumping. The Cyt*c*O was reconstituted in lipid vesicles and the pH dye phenol red was added to the outside of these vesicles to detect changes in proton concentration upon mixing with reduced cytochrome *c*. Valinomycin was added to deplete the electrical component of the electrochemical gradient (pumping, lower trace). Addition of the proton ionophore, CCCP resulted in total depletion of the electrochemical gradient across the membrane allowing detection of the net consumption of protons during enzymatic turnover (upper trace). Experimental conditions after mixing in a stopped-flow spectrophotometer: 0.25 μM vesicle reconstituted Cyt*c*O, 50 μM HEPES, pH 7.4, 45 mM KCl, 44 mM sucrose, 1 mM EDTA, 100 μM phenol red, 8 μM reduced cytochrome *c*, 2.5 μM valinomycin and 2.5 μM CCCP. The inside of the vesicles contained 0.1 M HEPES at pH 7.4.

As seen in Fig. 5, also with the Ser425Ala variant the **P**^**3**^ **→** **F**^**3**^ reaction rate was essentially pH independent in the measured pH range, i.e. the p*K*_a_ associated with this reaction was shifted to a value >10.5. In the context of the model outlined above, the structural change as a result of the Ser425Ala replacement may alter the Glu286 environment to modify the equilibrium constant between positions 1 and 2, resulting in an increased observed p*K*_a_. To explain that the Ser425Ala variant still pumps protons we speculate that in the mutant the ratio of the proton-transfer rates from Glu286 to the catalytic site, to the PLS and the structural relaxation ((*i*)-(*iii*) above) would be such that the stoichiometry of the proton pump would not be decreased (c.f. the Ser425Ala variant pumps protons). It should also be noted that the experiments described in this study were carried out in the absence of any electrochemical potential across the membrane. When present, this potential would presumably act to decrease proton transfer from Glu286 to the PLS more significantly than from Glu286 to the catalytic site because the former has a larger component perpendicular to the membrane. Consequently, it is possible that the Ser425Ala variant would display a smaller proton-pumping stoichiometry than the wild-type Cyt*c*O in the living cell. A variable proton pumping stoichiometry depending on the proton electrochemical gradient has been addressed in a recent theoretical study^12^.

Wikström and Verkhovsky suggested an alternative model to explain the decrease in the **P**^**3**^ **→** **F**^**3**^ rate at pH>9 with the wild-type Cyt*c*O. They assumed that proton uptake into the D pathway occurs via a proton “antenna”^62^ surrounding the D-pathway orifice and that the slowed proton uptake at high pH is due to exhaustion of protons from these groups. Furthermore, they explained the absence of the pH-dependence in the **P**^**3**^ **→** **F**^**3**^ rate with e.g. the Asn139Asp variant in terms of an altered equilibrium between different configurations of the Glu286 side chain^63^, in part based on the observation of differences in the structure of the structural variant^25^ and the wild-type Cyt*c*O. Because it is unlikely that the Ser425Ala replacement would result in changes at the D pathway orifice (because of the large distance), also the “alternative view” would explain the altered pH dependence (of the **P**^**3**^ **→** **F**^**3**^ reaction) in the Ser425Ala variant in terms of changes of the Glu286 configuration.

The discussion above is focused on the **P**^**3**^ **→** **F**^**3**^ reaction because this step is only associated with proton transfer through the D pathway and there is no interference from internal electron transfer. Also the **F**^**3**^ **→** **O**^**4**^ reaction involves proton transfer through the D pathway. However, this reaction is also linked to simultaneous electron transfer to the catalytic site, which complicates a detailed quantitative interpretation of the data because both electron and proton transfer determine the overall **F**^**3**^ **→** **O**^**4**^ rate^64^ (see also^65^).

To summarize, the structural data obtained earlier with both the mitochondrial and the *R. sphaeroides* Cyt*c*Os show significant structural changes of Ser425 and surrounding residues upon redox changes of heme *a*^8^,^21^,^22^,^23^,^24^. The data from the present study show that the Ser425Ala replacement results in altering specific reaction steps that are linked to proton pumping. In other words, the combined structural data and the functional data from this study point to a link between redox reactions and proton pumping. Our findings are also consistent with earlier studies using Fourier Transform Infrared Spectroscopy (FTIR) showing a link between redox and structural changes at Glu286^66^,^67^. The above-discussed model explains qualitatively these effects in terms of a link between changes at Ser425 and Glu286. However, the discussed changes at Glu286 are likely to comprise only part of the problem to describe the mechanism of proton pumping. As already noted above, the D pathway in the bacterial Cyt*c*O is used to transfer both the proton that is used in the reaction that drives the pump and to transfer the proton that is pumped across the membrane. This scenario requires control of the proton flux at two levels: (*a*) at Glu286 to control the timing of proton transfer to the catalytic site and the PLS, respectively, (*b*) at the PLS where the flux of the pumped protons across the membrane must be controlled (c.f. proton gating). Problem (*a*) requires control of the proton flux via Glu286, where the two proton-transfer trajectories branch^47^, while problem (*b*) requires control of the proton access to and from the PLS toward the *p* side.

Even though the above-described model (Scheme 1 and Equations 1, 2) is focused only on problem (*a*), proton gating at Glu286 and at the PLS are presumably tightly coupled. As briefly outlined in the Introduction section, results from theoretical studies indicate that the PLS is located around the heme *a*_3_ propionic acids and most likely involves also a cluster surrounding residues^14^. Structural changes in this protein segment, around propionate A of heme *a*_3_, have been suggested to take place upon reduction of heme *a*. This coupling may be important both for controlling protonation of the PLS and it’s proton connectivity to the two sides of the membrane^31^. Taken together, the earlier observations combined with those from the current study point to a link between redox changes at the cofactors, structural changes at Ser425, structural changes at Glu286 as well as at the putative PLS at the heme *a*_3_ propionic acids.

## Materials and Methods

### Site-directed mutagenesis, protein expression and purification

The Ser425 to Ala/Val/Thr mutations were introduced into the Cyt*c*O SUI-containing plasmid pJS3-SH, using the Quik-Change site-directed mutagenesis kit (Stratagene/Agilent technologies) as described in^68^. The amino-acid replacements were verified by sequencing. A DNA fragment containing the mutated site was introduced into the plasmid pRK-415, which holds SU I-III of *R. sphaeroides* Cyt*c*O. The pRK-415 plasmid was transformed into *E. coli* strain S-17 by electroporation and thereafter transferred to the *R. sphaeroides* strain JS100 by conjugation. For Cyt*c*O expression, *R. sphaeroides* cells were grown aerobically in shaker incubators at 30 °C in the dark. The inner membrane fraction was collected by ultracentrifugation and Cyt*c*O was solubilized in *n*-dodecyl β-D-maltoside (DDM). The Cyt*c*O (His-tagged) was purified using Ni^2+^-NTA affinity chromatography, essentially as described in^68^,^69^.

### Steady-state activity

A buffer composed of 50 mM K-P_i_ (pH 6.7), 0.1% DDM and 1.0 mg/ml asolectin, supplemented with 6 mM ascorbate, 670 μM *N*,*N*,*N′*,*N′*-tetramethyl-*p*-phenylenediamine (TMPD, used as an electron mediator) and 32 μM cytochrome *c* was added to the reaction chamber of a Clark-type O_2_ electrode (Hansatech instruments). Cyt*c*O at a concentration of 1 μM in a buffer containing 100 mM Hepes (pH 7.5) and 0.05% DDM was added to the reaction chamber to a final Cyt*c*O concentration of 7 nM. The oxygen-consumption rate during Cyt*c*O turnover was monitored. The steady-state activities of Ser425Ala, Ser425Val and Ser425Thr Cyt*c*Os were 70%, 19%, and 16% of that of the wild-type Cyt*c*O, respectively (~570 e^−^/s/Cyt*c*O).

### Measurement of the oxidation kinetics

Cytochrome *c* oxidase was diluted to ~10 μM in a buffer composed of 0.1 M HEPES (pH 7.5) and 0.05% DDM, and transferred to a locally manufactured Thunberg cuvette. The air in the cuvette was evacuated on a vacuum line and replaced by N_2_. The Cyt*c*O was reduced upon addition of ascorbate (2 mM) and hexaammin-rutheniumchloride (1 μM). The redox state of the enzyme was monitored spectrophotometrically and when full reduction was reached, the N_2_ atmosphere in the cuvette was exchanged for carbon monoxide (CO). CO binds to reduced heme *a*_3_, forming a CO-Cyt*c*O complex.

The CO-Cyt*c*O complex was mixed in about 10 ms (1:5, Cyt*c*O:O_2_-saturated solution) with an oxygen-saturated buffer (0.1 M HEPES (pH 7.5), 0.05% DDM, 0.1 mM EDTA) in a flow-flash apparatus (Applied Photophysics). About 200 ms after mixing, the CO ligand was dissociated from heme *a*_3_ by means of a 10 ns laser flash (Quantel Brilliant B, Nd-YAG, 532 nm), enabling oxygen to bind to heme *a*_3_. Absorbance changes associated with the reaction of the reduced Cyt*c*O with O_2_ were monitored as a function of time in a 10-mm cuvette (see also^70^).

The pH dependence of the reaction of the reduced Cyt*c*O with O_2_ was studied as follows^43^. The Cyt*c*O samples were diluted in 1 mM HEPES, 0.05% DDM, 100 μM EDTA at pH 7.5. The CO-Cyt*c*O complex was then rapidly mixed (1:5) with oxygen-saturated buffer solutions (100 mM), supplemented with 0.05% DDM, 0.1 mM EDTA, at different pH values: MES at pH 6 and 6.5; HEPES at pH 7, 7.5 and 8; BIS-TRIS propane, pH 7–9; CHES, pH 9 and 9.5; CAPS, pH 10, 10.5 and 11.

### Proton uptake measurements

Two PD-10 columns were used to exchange the buffer of the Cyt*c*O samples to 0.1 M KCl, pH 7.8, 0.05% DDM, 0.1 mM EDTA and the Cyt*c*O concentration was adjusted to 10 μM. The samples were then treated as described above in the section “ Measurement of the oxidation kinetics” with the exception that the concentration of hexaammin-ruthenium chloride was 0.2 μM. The oxygen-saturated solution contained 0.1 M KCl, pH 7.8, 0.05% DDM, 0.1 mM EDTA and 40 μM phenol red. Typically, 15 traces at 560 nm were collected and averaged. The measurements were then repeated in the presence of 0.1 M HEPES, pH 7.8, 0.05% DDM, 0.1 mM EDTA and these absorbance changes were subtracted from those obtained with the un-buffered solution yielding absorbance changes that were only associated with changes in the proton concentration. The exhaust from the mixing device (unbuffered solution) was collected under N_2_ atmosphere and then titrated with well-defined amounts of HCl to calibrate the absorbance changes to changes in proton concentration.

### Preparation of mixed valence CO-CytcO and flash photolysis measurements

First, the Cyt*c*O sample buffer was exchanged to 0.1 M BIS-TRIS propane, pH 8.5, 0.05% DDM, 0.1 mM EDTA on a PD-10 column and the Cyt*c*O sample at a concentration of 10 μM was transferred to a modified Thunberg cuvette. The atmosphere in the cuvette was exchanged for N_2_ and then to CO. During incubation of Cyt*c*O under pure CO atmosphere the catalytic site (heme *a*_*3*_/Cu_B_) becomes reduced and CO binds to the reduced heme *a*_3_. The Cyt*c*O with oxidized Cu_A_/heme *a* and reduced heme *a*_3_/Cu_B_ is referred to as the “mixed-valance state”. The CO ligand was dissociated by applying a 10 ns laser flash (Quantel Brilliant Nd:YAG, 532 nm) in a flash photolysis setup (Applied Photophysics), allowing electron transfer to be monitored at different single wavelengths. To improve the time resolution from μs to ns, the measuring light was pulsed (~1 ms pulses, which allowed measuring absorbance changes over ~500–600 μs).

### Reconstitution of cytochrome c oxidase in vesicles and proton pumping measurements

A lipid extract was made from soybean phosphatidylcholine (type II, Sigma) using diethyl ether followed by precipitation in acetone. The lipids were dried under a stream of N_2_. Small unilamellar vesicles (SUVs) were prepared by sonication of 40 mg/ml of the purified lipid extract in 0.1 M HEPES- at pH 7.4 with 2% sodium cholate, using a tip sonicator (Ultrasonic VCX 130, Chemical Instruments AB) at 40% energy output during 30 s on and 30 s off repetitive cycles for a total of 2 min/ml. After sonication, the vesicle solution was centrifuged at 1500 g for 20 min to remove titanium particles and lipid aggregates. Reconstitution of Cyt*c*O in the vesicles was performed using a modified version of the protocol described in^42^,^71^. Briefly, the purified Cyt*c*O was diluted to 6 μM in 0.1 M HEPES at pH 7.4 with 4% sodium cholate and was mixed with the lipid solution at a ratio of 1:1. Detergent was removed using Bio-Beads SM-2 Adsorbent (Bio-Rad Laboratories) allowing the Cyt*c*O to insert into the vesicles. The buffer was exchanged for 100 mM KCl, pH 7.4 using a PD-10 column. The Cyt*c*O turnover activity was measured as described above. This activity diminishes as an electrochemical proton gradient is built up across the membrane. Upon addition of ionophores (valinomycin and carbonyl cyanide 3-chlorophenylhydrazone (CCCP)) the gradient is removed and the activity increases again. The ratio of the rates obtained after and before addition of the ionophores is the respiratory-control ratio (RCR). Values in the range 4–7 were obtained.

The proton pumping measurements were performed as described in^72^. Briefly, liposome-reconstituted Cyt*c*O was diluted to 0.5 μM Cyt*c*O in 50 μM HEPES, pH 7.6, 45 mM KCl, 44 mM sucrose, 1 mM EDTA, 100 μM phenol red and mixed rapidly at a 1:1 ratio with 16 μM reduced cytochrome *c* in the same solution as the reconstituted enzyme in a stopped-flow spectrophotometer (Applied Photophysics). Absorbance changes of the pH sensitive dye were detected at 558,7 nm (the isosbestic point for cytochrome *c* oxidation). Valinomycin was added to the reconstituted vesicles at a concentration of 5 μM. In order to detect net consumption of protons during enzymatic turnover (for calibration purposes), the proton ionophore CCCP at 5 μM was finally added to the Cyt*c*O-vesicle solution.

## Additional Information

**How to cite this article**: Vilhjálmsdóttir, J. *et al.* Structural Changes and Proton Transfer in Cytochrome *c* Oxidase. *Sci. Rep.* **5**, 12047; doi: 10.1038/srep12047 (2015).
